# 3-Nitro-2-phenyl­chroman

**DOI:** 10.1107/S1600536812020806

**Published:** 2012-05-16

**Authors:** Pei-Hua Zhao, Er-Jun Hao, Ya-Qing Liu, Gui-Zhe Zhao

**Affiliations:** a Research Center for Engineering Technology of Polymeric Composites of Shanxi Province, College of Materials Science and Engineering, North University of China, Taiyuan 030051, People’s Republic of China; b Key Laboratory of Green Chemical Media and Reactions, Ministry of Education, College of Chemistry and Environmental Science, Henan Normal University, Xinxiang 453007, People’s Republic of China

## Abstract

In the title compound, C_15_H_13_NO_3_, the dihedral angle between the two aromatic rings is 79.25 (16)°.

## Related literature

For pharmaceutical and synthetic applications of compounds with a benzopyran framework, see: Horton *et al.* (2003 ▶); Murugesh *et al.* (1996 ▶); Engler *et al.* (1990 ▶). 
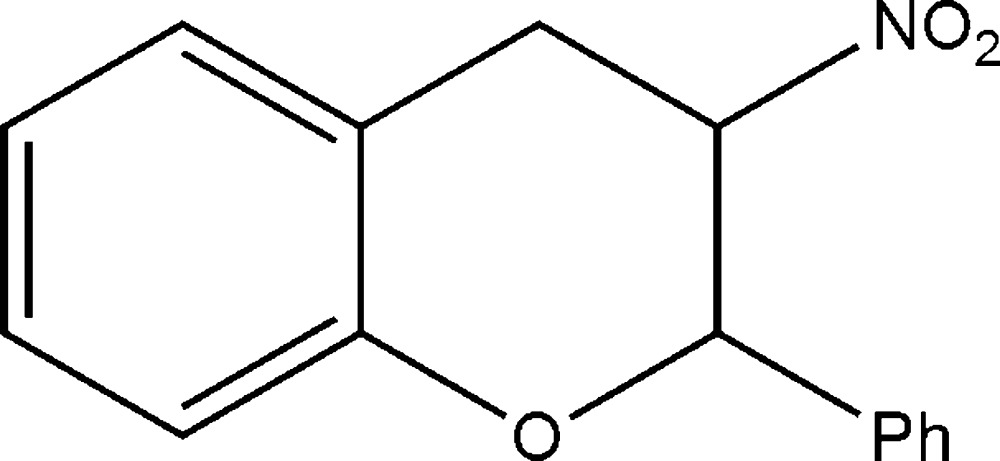


## Experimental

### 

#### Crystal data

C_15_H_13_NO_3_
*M* *_r_* = 255.26
Triclinic, 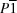

*a* = 5.3769 (11) Å
*b* = 10.105 (2) Å
*c* = 12.320 (3) Å
α = 70.85 (3)°
β = 82.89 (3)°
γ = 84.87 (3)°
*V* = 626.6 (2) Å^3^
*Z* = 2
Mo *K*α radiation
μ = 0.10 mm^−1^
*T* = 293 K
0.20 × 0.20 × 0.10 mm

#### Data collection

Rigaku Saturn diffractometer
Absorption correction: multi-scan (*CrystalClear*; Rigaku/MSC, 2005 ▶) *T*_min_ = 0.981, *T*_max_ = 0.991
5249 measured reflections
2205 independent reflections
912 reflections with *I* > 2σ(*I*)
*R*_int_ = 0.053

#### Refinement

*R*[*F* ^2^ > 2σ(*F* ^2^)] = 0.055
*wR*(*F* ^2^) = 0.152
*S* = 1.07
2205 reflections
172 parameters
H-atom parameters constrained
Δρ_max_ = 0.47 e Å^−3^
Δρ_min_ = −0.31 e Å^−3^

### 

Data collection: *CrystalClear* (Rigaku/MSC, 2005 ▶); cell refinement: *CrystalClear*; data reduction: *CrystalClear*; program(s) used to solve structure: *SHELXS97* (Sheldrick, 2008 ▶); program(s) used to refine structure: *SHELXL97* (Sheldrick, 2008 ▶); molecular graphics: *SHELXTL* (Sheldrick, 2008 ▶); software used to prepare material for publication: *SHELXTL*.

## Supplementary Material

Crystal structure: contains datablock(s) global, I. DOI: 10.1107/S1600536812020806/tk5093sup1.cif

Structure factors: contains datablock(s) I. DOI: 10.1107/S1600536812020806/tk5093Isup2.hkl

Supplementary material file. DOI: 10.1107/S1600536812020806/tk5093Isup3.cml

Additional supplementary materials:  crystallographic information; 3D view; checkCIF report

## Figures and Tables

**Fig. 1.**
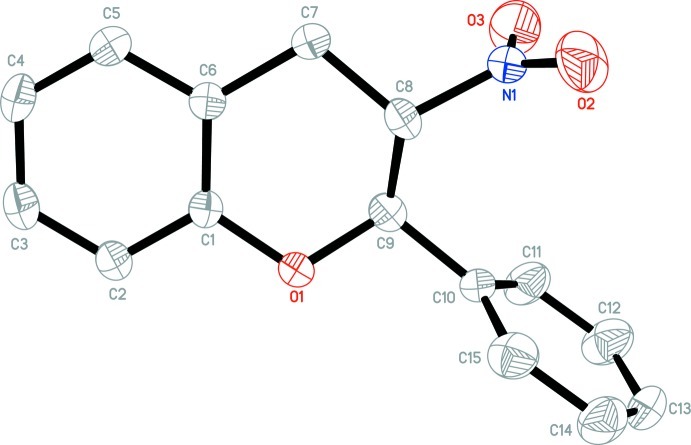
The molecular structure of (I). Displacement ellipsoids are drawn at the 30% probability level and H atoms are shown as small spheres of arbitrary radii.

## supplementary crystallographic information

### Comment

Compounds containing a benzopyran framework have anti-tumour, anti-bacterial and anti-inflammatory activities (Horton *et al.*, 2003). Additionally, they are also useful intermediates in the synthesis of complex natural products (Engler *et al.*,1990; Murugesh *et al.*, 1996). The title compound, a member of this class of compounds, was synthesised and characterised by X-ray crystallography.

As shown in Fig. 1, the crystal structure determination indicates that the dihedral angle between the two aromatic rings is 79.25 (16)°.

### Experimental

2-Phenyl-1-nitroethane (10.5 mmol), dimethyl amine hydrochloride (20 mmol), benzaldehyde (10.5 mmol), toluene (7.5 ml) and potassium fluoride (0.08 mmol) were taken in a 50 ml round bottomed flask fitted with a Dean-Stark water separator. The mixture was refluxed with stirring for 10 h. The solvent was removed from the reaction vessel to give a crude product. Chloroform (5 ml) and 0.2 *M* HCl (10 ml) were added to the crude material and the solution was heated on a water bath at 60 °C for 2 min under reduced pressure. The mixture was extracted with dichloroform. The organic extracts were dried over anhydrous magnesium sulfate. The residue was chromatographed on silica gel by eluting with EtOAc/pet. ether to give the desired product. Crystals of the title compound were obtained by slow evaporation of its dichloromethane/n-hexane solution at room temperature. ^1^N NMR (400 MHz, CDCl_3_, TMS): 7.43 (s, 5H), 7.24 (m, 2H), 7.02 (m, 2H), 5.45 (d, 1H, J = 8.0 Hz), 5.08 (m, 1H), 3.69 (dd, 1H, J = 9.2, 16.0 Hz), 3.35 (dd, 1H, J = 9.2, 16.0 Hz) p.p.m.. ^13^C NMR (100.6 MHz, CDCl_3_, TMS): 153.3, 135.8, 129.5, 129.4, 129.0, 128.5, 126.9, 122.0, 117.7, 117.0, 84.0, 78.0, 29.8 p.p.m..

### Refinement

The H atoms were positioned geometrically (C—H = 0.95–0.98 Å) and refined as riding with *U*_iso_(H) = 1.2*U*_eq_(C).

### Crystal data

**Table:**

C_15_H_13_NO_3_	*Z* = 2
*M**_r_* = 255.26	*F*(000) = 268
Triclinic, *P*1	*D*_x_ = 1.353 Mg m^−^^3^
Hall symbol: -P 1	Mo *K*α radiation, λ = 0.71073 Å
*a* = 5.3769 (11) Å	Cell parameters from 1554 reflections
*b* = 10.105 (2) Å	θ = 3.5–28.0°
*c* = 12.320 (3) Å	µ = 0.10 mm^−^^1^
α = 70.85 (3)°	*T* = 293 K
β = 82.89 (3)°	Prism, colourless
γ = 84.87 (3)°	0.20 × 0.20 × 0.10 mm
*V* = 626.6 (2) Å^3^	

### Data collection

**Table:**

Rigaku Saturn diffractometer	2205 independent reflections
Radiation source: rotating anode	912 reflections with *I* > 2σ(*I*)
Confocal monochromator	*R*_int_ = 0.053
ω scans	θ_max_ = 25.0°, θ_min_ = 3.2°
Absorption correction: multi-scan (*CrystalClear*; Rigaku/MSC, 2005)	*h* = −6→6
*T*_min_ = 0.981, *T*_max_ = 0.991	*k* = −11→10
5249 measured reflections	*l* = −14→14

### Refinement

**Table:**

Refinement on *F*^2^	Primary atom site location: structure-invariant direct methods
Least-squares matrix: full	Secondary atom site location: difference Fourier map
*R*[*F*^2^ > 2σ(*F*^2^)] = 0.055	Hydrogen site location: inferred from neighbouring sites
*wR*(*F*^2^) = 0.152	H-atom parameters constrained
*S* = 1.07	*w* = 1/[σ^2^(*F*_o_^2^) + (0.0551*P*)^2^] where *P* = (*F*_o_^2^ + 2*F*_c_^2^)/3
2205 reflections	(Δ/σ)_max_ < 0.001
172 parameters	Δρ_max_ = 0.47 e Å^−^^3^
0 restraints	Δρ_min_ = −0.31 e Å^−^^3^

### Special details

**Table:**

Geometry. All e.s.d.'s (except the e.s.d. in the dihedral angle between two l.s. planes) are estimated using the full covariance matrix. The cell e.s.d.'s are taken into account individually in the estimation of e.s.d.'s in distances, angles and torsion angles; correlations between e.s.d.'s in cell parameters are only used when they are defined by crystal symmetry. An approximate (isotropic) treatment of cell e.s.d.'s is used for estimating e.s.d.'s involving l.s. planes.
Refinement. Refinement of *F*^2^ against ALL reflections. The weighted *R*-factor *wR* and goodness of fit *S* are based on *F*^2^, conventional *R*-factors *R* are based on *F*, with *F* set to zero for negative *F*^2^. The threshold expression of *F*^2^ > σ(*F*^2^) is used only for calculating *R*-factors(gt) *etc*. and is not relevant to the choice of reflections for refinement. *R*-factors based on *F*^2^ are statistically about twice as large as those based on *F*, and *R*- factors based on ALL data will be even larger.

### Fractional atomic coordinates and isotropic or equivalent isotropic displacement parameters (Å^2^)

**Table:**

	*x*	*y*	*z*	*U*_iso_*/*U*_eq_	
O1	0.1296 (3)	0.85831 (18)	0.15383 (15)	0.0443 (5)	
O2	0.6208 (5)	0.6744 (3)	0.4385 (2)	0.0835 (8)	
O3	0.2445 (5)	0.6648 (3)	0.5113 (2)	0.0841 (9)	
N1	0.4007 (6)	0.6773 (2)	0.4307 (2)	0.0491 (7)	
C1	0.0165 (5)	0.7495 (3)	0.1373 (2)	0.0399 (7)	
C2	−0.1198 (6)	0.7845 (3)	0.0429 (2)	0.0512 (8)	
H2	−0.1301	0.8766	−0.0060	0.061*	
C3	−0.2397 (6)	0.6827 (4)	0.0218 (3)	0.0644 (10)	
H3	−0.3332	0.7058	−0.0412	0.077*	
C4	−0.2215 (7)	0.5453 (3)	0.0943 (3)	0.0667 (10)	
H4	−0.3032	0.4759	0.0805	0.080*	
C5	−0.0821 (6)	0.5123 (3)	0.1865 (3)	0.0542 (9)	
H5	−0.0692	0.4197	0.2344	0.065*	
C6	0.0406 (5)	0.6137 (3)	0.2101 (2)	0.0401 (7)	
C7	0.1895 (5)	0.5749 (3)	0.3130 (2)	0.0456 (8)	
H7A	0.0780	0.5414	0.3834	0.055*	
H7B	0.3127	0.4998	0.3097	0.055*	
C8	0.3187 (7)	0.6980 (3)	0.3148 (3)	0.0594 (9)	
H8	0.4734	0.7018	0.2629	0.071*	
C9	0.1824 (7)	0.8331 (3)	0.2685 (3)	0.0637 (10)	
H9	0.0198	0.8268	0.3151	0.076*	
C10	0.3006 (6)	0.9590 (3)	0.2757 (3)	0.0467 (8)	
C11	0.1942 (6)	1.0227 (3)	0.3531 (3)	0.0658 (10)	
H11	0.0462	0.9899	0.3978	0.079*	
C12	0.3002 (8)	1.1345 (4)	0.3669 (3)	0.0782 (12)	
H12	0.2248	1.1764	0.4205	0.094*	
C13	0.5127 (7)	1.1822 (3)	0.3026 (3)	0.0679 (11)	
H13	0.5866	1.2567	0.3125	0.081*	
C14	0.6212 (6)	1.1230 (4)	0.2231 (3)	0.0676 (10)	
H14	0.7674	1.1577	0.1778	0.081*	
C15	0.5138 (7)	1.0110 (3)	0.2099 (3)	0.0606 (9)	
H15	0.5882	0.9706	0.1552	0.073*	

### Atomic displacement parameters (Å^2^)

**Table:**

	*U*^11^	*U*^22^	*U*^33^	*U*^12^	*U*^13^	*U*^23^
O1	0.0569 (13)	0.0382 (11)	0.0365 (11)	−0.0084 (10)	−0.0131 (10)	−0.0056 (9)
O2	0.0496 (16)	0.101 (2)	0.094 (2)	0.0026 (13)	−0.0252 (15)	−0.0196 (15)
O3	0.094 (2)	0.103 (2)	0.0542 (15)	−0.0355 (16)	0.0116 (15)	−0.0226 (14)
N1	0.0566 (19)	0.0415 (15)	0.0463 (17)	−0.0090 (13)	−0.0147 (15)	−0.0052 (12)
C1	0.0473 (18)	0.0380 (17)	0.0373 (17)	−0.0044 (14)	−0.0070 (14)	−0.0143 (14)
C2	0.070 (2)	0.0444 (17)	0.0413 (18)	−0.0029 (16)	−0.0167 (16)	−0.0128 (15)
C3	0.083 (3)	0.066 (2)	0.052 (2)	−0.006 (2)	−0.0293 (19)	−0.0204 (19)
C4	0.087 (3)	0.058 (2)	0.069 (2)	−0.0154 (19)	−0.028 (2)	−0.0283 (19)
C5	0.067 (2)	0.0424 (18)	0.053 (2)	−0.0135 (16)	−0.0101 (18)	−0.0110 (15)
C6	0.0431 (18)	0.0395 (16)	0.0384 (17)	−0.0048 (14)	−0.0057 (14)	−0.0120 (14)
C7	0.0541 (19)	0.0345 (16)	0.0472 (18)	−0.0075 (14)	−0.0133 (15)	−0.0074 (14)
C8	0.084 (3)	0.048 (2)	0.049 (2)	−0.0038 (18)	−0.0345 (18)	−0.0092 (16)
C9	0.097 (3)	0.045 (2)	0.051 (2)	−0.0102 (19)	−0.032 (2)	−0.0076 (16)
C10	0.060 (2)	0.0340 (16)	0.0442 (18)	−0.0099 (16)	−0.0170 (17)	−0.0037 (14)
C11	0.061 (2)	0.065 (2)	0.070 (2)	−0.0217 (19)	0.008 (2)	−0.019 (2)
C12	0.106 (3)	0.064 (2)	0.072 (3)	−0.022 (2)	0.012 (2)	−0.034 (2)
C13	0.082 (3)	0.056 (2)	0.071 (2)	−0.027 (2)	−0.018 (2)	−0.018 (2)
C14	0.046 (2)	0.068 (2)	0.081 (3)	−0.0128 (19)	−0.003 (2)	−0.013 (2)
C15	0.074 (3)	0.049 (2)	0.060 (2)	0.0029 (19)	−0.003 (2)	−0.0217 (17)

### Geometric parameters (Å, º)

**Table:**

O1—C1	1.385 (3)	C7—H7A	0.9700
O1—C9	1.411 (3)	C7—H7B	0.9700
O2—N1	1.196 (3)	C8—C9	1.463 (4)
O3—N1	1.199 (3)	C8—H8	0.9800
N1—C8	1.490 (3)	C9—C10	1.505 (4)
C1—C6	1.376 (4)	C9—H9	0.9800
C1—C2	1.381 (4)	C10—C15	1.359 (4)
C2—C3	1.370 (4)	C10—C11	1.361 (4)
C2—H2	0.9300	C11—C12	1.376 (4)
C3—C4	1.385 (4)	C11—H11	0.9300
C3—H3	0.9300	C12—C13	1.338 (5)
C4—C5	1.371 (4)	C12—H12	0.9300
C4—H4	0.9300	C13—C14	1.356 (5)
C5—C6	1.391 (4)	C13—H13	0.9300
C5—H5	0.9300	C14—C15	1.379 (4)
C6—C7	1.507 (4)	C14—H14	0.9300
C7—C8	1.486 (4)	C15—H15	0.9300
			
C1—O1—C9	114.2 (2)	C9—C8—N1	112.6 (3)
O2—N1—O3	123.1 (3)	C7—C8—N1	111.7 (2)
O2—N1—C8	118.0 (3)	C9—C8—H8	105.7
O3—N1—C8	118.9 (3)	C7—C8—H8	105.7
C6—C1—C2	121.8 (3)	N1—C8—H8	105.7
C6—C1—O1	121.8 (2)	O1—C9—C8	112.2 (2)
C2—C1—O1	116.3 (3)	O1—C9—C10	109.2 (2)
C3—C2—C1	119.7 (3)	C8—C9—C10	116.0 (3)
C3—C2—H2	120.2	O1—C9—H9	106.3
C1—C2—H2	120.2	C8—C9—H9	106.3
C2—C3—C4	119.9 (3)	C10—C9—H9	106.3
C2—C3—H3	120.1	C15—C10—C11	117.9 (3)
C4—C3—H3	120.1	C15—C10—C9	122.9 (3)
C5—C4—C3	119.6 (3)	C11—C10—C9	119.2 (3)
C5—C4—H4	120.2	C10—C11—C12	121.6 (3)
C3—C4—H4	120.2	C10—C11—H11	119.2
C4—C5—C6	121.7 (3)	C12—C11—H11	119.2
C4—C5—H5	119.2	C13—C12—C11	119.4 (3)
C6—C5—H5	119.2	C13—C12—H12	120.3
C1—C6—C5	117.4 (3)	C11—C12—H12	120.3
C1—C6—C7	122.1 (2)	C12—C13—C14	120.6 (3)
C5—C6—C7	120.6 (3)	C12—C13—H13	119.7
C8—C7—C6	110.6 (2)	C14—C13—H13	119.7
C8—C7—H7A	109.5	C13—C14—C15	119.7 (3)
C6—C7—H7A	109.5	C13—C14—H14	120.2
C8—C7—H7B	109.5	C15—C14—H14	120.2
C6—C7—H7B	109.5	C10—C15—C14	120.8 (3)
H7A—C7—H7B	108.1	C10—C15—H15	119.6
C9—C8—C7	114.6 (3)	C14—C15—H15	119.6
			
C9—O1—C1—C6	−23.5 (4)	O3—N1—C8—C7	−63.3 (4)
C9—O1—C1—C2	157.1 (3)	C1—O1—C9—C8	50.9 (4)
C6—C1—C2—C3	1.4 (4)	C1—O1—C9—C10	−179.1 (2)
O1—C1—C2—C3	−179.2 (3)	C7—C8—C9—O1	−57.3 (4)
C1—C2—C3—C4	−0.7 (5)	N1—C8—C9—O1	173.5 (3)
C2—C3—C4—C5	−0.3 (5)	C7—C8—C9—C10	176.4 (3)
C3—C4—C5—C6	0.6 (5)	N1—C8—C9—C10	47.2 (4)
C2—C1—C6—C5	−1.1 (4)	O1—C9—C10—C15	−57.1 (4)
O1—C1—C6—C5	179.5 (2)	C8—C9—C10—C15	70.7 (4)
C2—C1—C6—C7	179.9 (3)	O1—C9—C10—C11	124.4 (3)
O1—C1—C6—C7	0.5 (4)	C8—C9—C10—C11	−107.8 (4)
C4—C5—C6—C1	0.1 (4)	C15—C10—C11—C12	−1.4 (5)
C4—C5—C6—C7	179.1 (3)	C9—C10—C11—C12	177.2 (3)
C1—C6—C7—C8	−5.5 (4)	C10—C11—C12—C13	0.2 (6)
C5—C6—C7—C8	175.5 (3)	C11—C12—C13—C14	1.1 (6)
C6—C7—C8—C9	32.9 (4)	C12—C13—C14—C15	−1.1 (6)
C6—C7—C8—N1	162.5 (3)	C11—C10—C15—C14	1.4 (5)
O2—N1—C8—C9	−112.4 (3)	C9—C10—C15—C14	−177.2 (3)
O3—N1—C8—C9	67.4 (4)	C13—C14—C15—C10	−0.1 (5)
O2—N1—C8—C7	116.9 (3)		
